# Practical Points

**Published:** 1904-02

**Authors:** 


### PRACTICAL POINTS.

Porcelain Inlay Non-Stainable.—Tobacco will not stain a porcelain inlay as it will the tooth in which the inlay is inserted—so say tobacco chewers.

Treating a Child.—A man makes an appointment with a child lacking in everything that goes to make a good patient, and puts his very life into the operation and the attempt to educate that child for future operation. The results are not beautiful to look upon, nor are they of much value to the tooth. The operator leaves the chair sick at heart, discouraged and physically worn. You, my friends, may see this case next fall. As you look upon this faulty piece of work, and may be the only one you will ever see from the man across the way, you will be able to put yourself exactly at the right point of observation to see this picture as a whole, or will you see the failure through glasses that are colored? Will you say to the child or its mother, and perhaps her friends, that the work is a failure and must all be done over? Will you take this child, now older and perhaps improved as a patient by the lessons so patiently taught by the man across the way, and perhaps in many ways better able to meet you half way to the end that a better operation may result? Will you steal the life-blood of your neighbor and use it without credit to him? Will you classify him by the result of this operation, by the clothes he wears, or the style of sign he displays upon his office window?—*Review.*

Repairing Amalgam Fillings.—In case a portion of the tooth has broken away from an otherwise perfectly good amalgam filing, clean the surface of the filling with a cross-bur, leaving it in polished ridges. Wipe over the surface with phosphoric acid (the liquid of Harvard cement is good). Then burnish soft fresh amalgam on the prepared surface, with which it will combine at once; then build up to the required shape. Saliva has no weakening effect on the joint.—*British Dental Jour.*

How to Extract Broken-Down Roots of Bicuspids and Laterals.—For extracting roots of lower biscuspids and upper laterals, where decay has extended to the process or beyond it, where the elevator could not be used, try a screw and you will be surprised at the result. Wash debris out of the root, drill slightly into the sound portion, following the canal; put in your screw tightly, then extract by grasping the screw with narrow-beaked root-forceps, giving a straight pull; do not luxate.—*Summary.*

The Best Age for Regulating.—Generally speaking the younger the age at which regulation is undertaken the better. Teeth should be regulated at the time they are erupting; they are not clasped fully at at that time by the alveolus, but are in large open cells and are moved with the utmost readiness; this is illustrated by the fact of the permanent teeth being so easily deflected by the temporary teeth.—Mr. Badcock, *British Dental Journal.*

Polishing Porcelain.—After grinding thep orcelain, go over the surface with a sandpaper disk, following with a cuttlefish disk. Then take oxide of tin polishing putty, and with wooden points, known as Barker's porous polishers, as beautiful a surface may be given to porcelain as can be imparted to it by fusing. By this method one may have the assurance that a perfect polish may be given inlays without fusing again,—*Review.*

Cotton as a Wedge.—Cotton seems to be coming into favor again in slow wedging, but some who use it fail to distinguish between absorbent cotton and the old fashioned cotton batting. This latter comes to the dentist frequently as packing in small boxes. It is oily and highly elastic; it takes up an abundance of moisture in a little time and swells to generous proportions. This is the kind that should be used. Absorbent cotton absorbs—that's wat it's for; it doesn't swell; it is inelastic. As a wedging material it is practically worthless.—Office and Laboratory.

It has been shown by careful experimental research that when arsenous acid is applied to the dental pulp a considerable portion is absorbed by the proteid elements of the pulp tissue and by the hemo-globin of the vascular supply to the organ. These proteid compounds of arsenic are in relatively weak chemical combination, and in the course of a short time, if the poisoned pulp tissue be not promptly removed, the arsenic is liberated by the breaking down of its proteid combination first formed in the pulp, and is diffused throughout the structure of the root until it sets up an arsenical necrosis in the pericementum.—*Ex. Editorial Cosmos.*

To Facilitate Taking Plaster Impressions for Partial Dentures.—Use a flat bottom tray and before putting the plaster in place fit in two pieces of metal plate bent to right angles so that the horizontal part lies on the bottom of the tray and the perpendicular part will stand upright and extending mesio-distally. These upright parts will remain in the plaster impression and will serve as line of cleavage in breaking the impression away from the teeth after the tray is removed.—Dr. E. L. Townsend, *Pacific Gazette.*

To Make a Handy Mouth Blow-Pipe.—Obtain an od brass bicycle pump and remove the plunger. Unscrew the cap and insert the end of a small mouth blow-pipe (about four inches long) in the hole in the cap as far as it will go. Mark it, cut off the small projecting end and solder to the cap. Now make a hole in the opposite end of the pump large enough to admit the remaining end of the pipe and attach with solder. Obtain some brass wire gauze, roll it with cotton and pack loosely in the pump chamber. Saturate the gauze with gasoline or alcohol and use with an alcohol or gas flame. You will find this a very convenient appliance for soldering at the chair and waxing up cases. Make one when you have time and you will not regret it.—Dr. C. J. Hadley, *Register.*

Dentists Better Trained.—It is no idle boast to say that dental graduates are better trained today than ever before, and with the new requirements of a four years' course there will come still greater improvements in methods and results, notwithstanding the statement a prominent State Board Examiner made recently that the dental colleges had taken a foolish position in increasing the length of the course. We believe that time will demonstrate that the college standard is safer than any State Board.—Ex. Editorial, *Register.*

*The Treatment of Certain Malignant Growths by Excision of the External Carotids,* by Robert H. M. Dawbarn, M. D., Professor of Surgery and Surgical Anatomy in the New York Polyclinic Medical School and Hospital, Visiting Surgeon to the City Hospital, New York, etc. (The Samuel D. Cross prize essay.) 8 vo., pages xiii–192. Extra cloth, price $2.00, net, delivered. Philadaphia, Pa. F. A. Davis Company, Publishers, 1914–16 Cherry Street.

This is a book of some 200 pages, which is an exceptionally good work. The fact that it received the S. D. Gross prize of $1,000.00 in 1902 is a criterion of its superior excellence. It is a book that should be in every medical library and one that can be read with profit by the dentist as well.

*A Text Book of Practical Gynecology* for Practitioners and Students, by D. Todd Gilliam, M. D., Professor of Gynecology in Starling Medical College, Columbus, O.; Gynecoogist to St. Anthony and St. Francis Hospitals, Columbus, O.; Fellow of the American Medical Association of Obstetricisan and Gynecologists; Member of the American Medical Association of the Ninth International Medical Congress and of the Pan-American Medical Congress; Honorary Member of the Northwestern Medical Association, Consulting Gynecologist to Park View Sanitarium, etc. Royal octavo, pages xvi–634. Illustrated with 350 engravings, a colored frontispiece and U full page half-tone plates. Extra cloth, $4.00 net, Half-Russa, $5.00 net, delivered. Philadelphia, F. A. Davis Company, Publishers, 1914–16 Cherry Street.

A practical and therefore valuable book for the busy medical practitioner. Bibliographic references and citations of authorities have been practically dispensed with, moot questions and effete matter being excluded altogether. It is a book compiled largely from the author's personal experience of cases, and is arranged and worded in a pain connected way as to make it most valuable for reference and study.

The illustrations are of a better grade than those annually found in medical works.

Treatment of Diseased Bone—G. W. Cook.—For diseased bone conditions I use equal parts of carbolic acid and sulphuric acid, which is a bland preparation. It is not so irritant as is sulphuric acid alone. It is more antiseptic, and has more affinity for the micro-organisms involved in the disease than has any other agent we can use outside of chinosol.—*Review.*

Where Cocaine is Serviceable.—J. G. Reid.—The one place where cocaine is a most serviceable agent is in the removal of portions of necrosed bone from a small cavity where I want to pack the tissues for a few days. I find that the application of cocaine for a few moments enables me to introduce the packing with much more success and comfort to my patient. I also use cocaine before introducing arsenic to the pulp. I do that in almost every case. I invariably have my rubber dam in position, and am not flooding the mouth with any uncertain percentage of cocaine. I find that I have less trouble with the after-results of arsenic by such a procedure.—*Extract Review.*

Where Cocaine is Most Dangerous—E. R. Carpenter.—From all I have found written on the subject of its toxic effect, I gather that the most dangerous use of cocaine is to inject it into deep-seated cavities, such as the bladder, sinuses or abscesses.—*Extract Review.*

How to Use Cocaine for Gums and Pericementum—E. R. Carpenter.—I pack powdered crystals of cocaine under the free margin of the gum against the pericementum at points where both jaws of the clamp grasp the root, and in a minute to a minute and a half I can press the gum away from the margin of the cavity, and force the clamp to place with but little or almost no pain to the patient. In this procedure I will add that the rubber dam is applied first, and the hole of the rubber dam encircling the tooth to be thus anesthetised is drawn to one side by the forefinger, enlarging it just enough to expose the edge of the gum, when cocaine can be readily packed between the gum and pericementum without th danger of getting it into the mouth and having the saliva carry it to the tongue and throat. Should the cavity be so far beneath the gum as to preclude the possibility of forcing the gum out of the way, I unhesitatingly clamp well on to the gum itself, and dissect away the exposed flap with a sharp spoon excavator, dipping same into a 95 per cent. solution of carbolic acid before using. The acid sears over the edge of wound, thus inhibiting the action of micro-organisms upon the freshly cut surfaces after the clamp has been removed.—*Extract Review.*

Use of the Ferrule in Crowning—H. C. Register.—I do not hesitate to say that there are as many, if not more, roots destroying by crowning than are saved by it, because of the production of irritation, which induces gingivitis leading to pericementitis and the other ills that follow irritation. The ferrule should be used with extreme care. I very rarely, except in exceptional cases, let it run up above the gum at all. I prefer a plate of gold or platinum well fitted to the face of the root. Through this is passed the post of a Logan crown, which has its labial edge nicely adapted to the labial part of the plate, while the lingual edge of the porcelain is well ground away, leaving a space between the crown and the plate. After being satisfied that everything is as correct as one can make it, simply catch it on the inside or outside with a quantity of phosphate of zinc. I use petroid for this. If using a gold plate, fill in the space with low-fusing porcelain body; bake it in the furnace, and it comes out a perfectly solid tooth with a little gold or platina termination, and the adaptation is exact. A plain plate tooth may be used if preferred. When in position on the root you have not got the ring or ferrule under the gum to act as an irritant. The festoon of the gingiva will become normal. The chief use of the ferrule is to prevent the root from splitting.—*Extract International.*

Pulp Anesthesia Through the Peridental Membrane—E. C. Carpenter.—I am convinced after six years of close observation in daily practice that in thus cocainizing the peridental membrane the effect of the anesthesia is, to a considerable degree, carried to the pulp of the tooth. Since I have taken special precaution to anesthetize the peridental membrane, I observe that in fully nine-tenths of such cavities I can excavate with much less pain than ever before, and in many cases without pain at all. Thus you have the results of my clinical experience.—*Extract Review.*

Treatment of Abscessed Deciduous Teeth—C. N. Johnson has advocated the following: Cleanse canals as thoroughly as possible. Fill pulp-chambers with cotton saturated with oil of cloves, and by means of unvulcanized rubber exert pressure on cotton until oil of cloves is forced through sinus. Then flood cavity with solution gutta-percha in eucalyptus and force temporary stopping into each canal until the eucalyptus appears at fistulous opening. Will rarely give any further trouble.

Trimming of Lower Plates.—J. E. Line.—I have had trouble with lower dentures, much of it due to the over-extension of the plate. Many years ago a dentist who always made gold plates told him that the best rule is to cut away all of the plate you dare to, and then to cut away just a little more. The plate does not require the extension to hold it in place, and on the other hand it furnishes a lever, so to speak, by which the plate will be lifted by the movements of the mouth from the place where it should remain.—*Cosmos.*

Glass for Bracket and Cabinet.—J. H. More.—For years I have had my bracket covered, and where convenient, the drawers of my cabinet lined with glass instead of felt. This makes conditions more aseptic.—*Cosmos.*

Care in Use of Broaches.—R. C. Young.—In treating a tooth which has two or more roots, never use the same broach in different roots without cleansing it, for it may be that all the roots are not septic—abscesses are often confined to one root—and if the instrument be used in first one and then the other it is more than possible to carry infection into the other roots. Of course we would not allow the saliva under any circumstances to enter the cavity.—*Cosmos.*

To Prevent Soreness of Gum from the Application of Clamps to Teeth.—The soreness which follows the application of an ill-fitting clamp can be prevented in a great majority of cases by brushing the gum around the neck of the tooth with tincture of calendula. This preparation has the valuable property of preventing soreness and suppuration and of inducing the rapid development of healthy granulations.—*Cosmos.*

Guaiacol in the Extraction of Teeth.—B. Marichal.—Since the anesthetic properties of guaiacol became known, the idea was conceived of using this agent in dental surgery, and Dr. Marichal has obtained very satisfactory results with the use of the formula suggested by Dr. André, which is as follows:

℞—Guaiacol, chemically pure, 1 gram;
Olive oil sterilized and neutralized, q. s. to make 10 c.c. M.
Sig.—For hypodermic injection, each cubic centimeter containing 10 centigrams of guaiacol.

In one hundred extractions, Dr. André observed that the effect of guaiacol is at least equal to that of cocaine, and that in certain cases in which the cocaine does not produce the desired effect, as in periostitis, perialveolar abscess, and radicular cysts, guaiacol produces perfect analgesia—*analgesia,* as sensibility to touch is preserved, the sense of pain being the only sensation destroyed. This agent, then, is not a true anesthetic, but an analgesic.

Guaiacol has also produced favorable results in the treatment of pulpitis, the pain having subsided through the application of the drug in the carious cavity, without the necessity of opening the pulp chamber. The hypodermic injections of guaiacol are made in the same way as those of cocaine; the quantity injected is generally a cubic centimeter, and from three to five minutes should elapse between the injection and the extraction. Dr. Marichal recommends the use of this agent and asks his colleagues to test its efficiency. It being entirely non-toxic, and not caustic, its use as an agent for the painless extraction of teeth should become very general.—*Cosmos.*

Drying Pulp Cavities—W. H. Weaver.—To dry the dental tubuli after injecting a preparation or solution and removing the pulp at once, use a 50 per cent. solution of alcohol, increasing strength 10 per cent. every time and drying, until absolute alcohol is used the last time. It leaves vacant every space. It is immediately dried, so far as can be made so by the use of any preparation. We all know that alcohol is the great absorbent of moisture.—*Dental World.*

Die and Counter-Die of Mellotte's Metal.—Dr. A. H. Davis, in the Atlanta *Dental Journal,* recommends painting the surface of the die with a thin coat of whiting and water to prevent adhesion. It is then dried with a blast from the blowpipe. The counter-die may be poured reasonably hot. The parts separate readily.

[If No. 4 tinfoil be pasted over the die and pressed closely in contact with a soft piece of rubber and then dried, the die and counterdie will separate readily. If another coat of the paste be put over the tin next to the counterdie, the tin will not adhere to the counterdie. If this be not done the composition of the metal will be gradually changed by the addition of the tin. The paste used is ordinary paste sold at stationery stores.—McCLAIN.]—*International.*

INEXPENSIVE BUT EFFECTIVE STERILIZER.—R. C. YOUNG.—Now for the instruments—forceps, excavators, burs, knives, clamps, etc. A sterilizer can be made for a couple of dollars which will do just as efficient work as one that costs ten. Any tinner can manufacture one: A tin pan 6x12 inches, 4 in. deep, with a cover to fit close, a piece of perforated tin, with a handle on each end, which will fit nicely inside like a second bottom. Upon this the articles to be sterilized are to be placed; a solution of ordinary cooking soda, sodium bicarbonate, about two-thirds fills the pan, and it is placed over a Bunsen burner, or a coal-oil lamp used for the vulcanizer, to boil five or ten minutes; lift the false bottom out, and the heat of the instruments will dry them off in a few moments. The soda in the water prevents rusting; of course wooden-handled instruments cannot be boiled.

Before placing burs in the sterilizer, brush them off with a wet tooth-brush, or a brush of similar shape.—*Cosmos.*

VULCANIZATION OF LARGE PROSTHETIC PIECES.—DR. CLAUDE MARTIN vulcanizes large prosthetic pieces at a temperature of 311 deg. F. for two hours, and never has to record cases of porosity.—*Australian Journal.*

SHAPING THE IMPRESSION.—DR. BACHMAN says that if the tongue be thrust out while the plaster is still soft, and the mouth twisted in every direction, the impression will be so shaped that the plate will only cover so much as is absolutely necessary and there will be nothing to cut away.—*Cosmos.*

TAKING IMPRESSIONS TO AVOID SHRINKAGE.—DR. FAXON.—There is a question whether a misfitting plate is not due many times to the shrinkage of the plaster in taking the impression wholly of plaster, which leaves an uneven thickness of the plaster and therefore an uneven shrinkage, and in filling the impression the same trouble would occur. For the last few years it has been my method to form a tray of modeling compound and then run in a thin layer of plaster. In that process the plaster gives a very thin layer in which there would be absolutely no shrinkage. In filling that impression I add about one-fourth marble dust to the plaster, which I believe will bring the shrinkage to the minimum.—*Cosmos.*

